# Presence of *Helicobacter* Species in Gastric Mucosa of Human Patients and Outcome of *Helicobacter* Eradication Treatment

**DOI:** 10.3390/jpm12020181

**Published:** 2022-01-29

**Authors:** Rita Matos, Emily Taillieu, Sofie De Bruyckere, Chloë De Witte, Alexandra Rêma, Hugo Santos-Sousa, Jorge Nogueiro, Celso A. Reis, Fátima Carneiro, Freddy Haesebrouck, Irina Amorim, Fátima Gärtner

**Affiliations:** 1 Instituto de Investigação e Inovação em Saúde (i3S), Universidade do Porto, 4200-135 Porto, Portugal; ritam@ipatimup.pt (R.M.); h.santos.sousa@gmail.com (H.S.-S.); celsor@ipatimup.pt (C.A.R.); fcarneiro@ipatimup.pt (F.C.); fgartner@ipatimup.pt (F.G.); 2 Institute of Pathology and Molecular Immunology, University of Porto (IPATIMUP), 4200-135 Porto, Portugal; 3 Instituto de Ciências Biomédicas Abel Salazar, Universidade of Porto (ICBAS), 4050-313 Porto, Portugal; alexandra.rema@gmail.com; 4 Department of Pathobiology, Pharmacology and Zoological Medicine, Faculty of Veterinary Medicine, Ghent University, 9000 Ghent, Belgium; Emily.Taillieu@UGent.be (E.T.); Sofie.DeBruyckere@UGent.be (S.D.B.); Chloe.DeWitte@UGent.be (C.D.W.); Freddy.Haesebrouck@UGent.be (F.H.); 5 Serviço de Cirurgia Geral, Centro Hospitalar Universitário São João, 4200-319 Porto, Portugal; nogueiro.jorge@gmail.com; 6 Faculty of Medicine, University of Porto (FMUP), 4200-319 Porto, Portugal; 7 Serviço de Anatomia Patológica, Centro Hospitalar Universitário São João, 4200-319 Porto, Portugal

**Keywords:** *Helicobacter*, NHPH, human stomach, gastric disease, eradication treatment

## Abstract

The genus *Helicobacter* is composed of bacteria that colonize both the human and animal gastrointestinal tract. *Helicobacter pylori* infects half of the world’s population, causing various disorders, such as gastritis, duodenitis and gastric cancer. Additionally, non-*Helicobacter pylori Helicobacter* species (NHPH) are commonly found in the stomach of pigs, dogs and cats. Most of these species have zoonotic potential and prevalence rates of 0.2–6.0%, and have been described in human patients suffering from gastric disorders undergoing a gastric biopsy. The aim of this study was to determine the occurrence of *Helicobacter* spp. in the stomach of patients with gastric cancer (*n* = 17) and obese (*n* = 63) patients. Furthermore, the outcome of the *Helicobacter* eradication treatment and the current infection status was evaluated. Overall, based on the genus-specific PCR followed by sequencing, DNA from *Helicobacter* spp. was detected in 46.3% of the patients, including single infections with *H. pylori* in 43.8% of the patients and mixed infections with *H. pylori* and canine- or feline-associated *H. felis* in 2.5%. About 32.5% of the patients had been subjected to previous *Helicobacter* eradication therapy and the triple standard therapy was the most frequent scheme (42.3%). In 48.0% of the patients who received eradication treatment, bacteria were still detected, including one mixed infection. In 23.1% of the patients who reported that a subsequent test had been performed to confirm the elimination of the bacteria, *Helicobacter* were still detected. In conclusion, although in a smaller percentage, NHPH may also be present in the human stomach. Thus, specific NHPH screening should be included in the diagnostic routine. The continued presence of *H. pylori* in the stomach of patients recently subjected to eradication schemes raises questions about the efficacy of the current *Helicobacter* treatments.

## 1. Introduction

The *Helicobacter* genus consists of a group of Gram-negative, motile bacteria, colonizing the stomach and intestinal tract of humans and animals [1]. *Helicobacter pylori* (*H. pylori*) is the most common gastric *Helicobacter* species, with an average worldwide prevalence of 58%, and is considered a major public health issue [2,3,4]. Infection is usually acquired during childhood, and although the majority of the individuals remain asymptomatic, some develop gastric disorders, of which chronic gastritis is the most frequently described pathology [1,2,4,5]. In the majority of the cases, the gastritis is superficial, but progression to gastric or duodenal ulcers (10–20%) and/or gastric (adeno)carcinoma (1–2%) can also occur [4,5]. As such, *H. pylori* is considered a Group I carcinogen by the International Agency for Research on Cancer [4,6,7]. Multiple studies have shown that *H. pylori* eradication reduces gastric inflammation, thereby (i) preventing the progression towards pre-malignant lesions, (ii) decreasing the incidence of gastric carcinoma [7,8,9], and [10] improving corpus gastritis and dysplasia lesions [10]. Nevertheless, treatment efficacy has been declining in the last decade due to several factors such as side effects, including increasing antimicrobial resistance and the high costs of the antibiotic treatments [11].

Besides *H. pylori,* other gastric helicobacters, named non-*Helicobacter pylori* helicobacters (NHPH), have been described colonizing both the human and animal stomach [12,13,14,15]. So far, *H. suis, H. heilmannii, H. ailurogastricus, H. felis, H. salomonis* and *H. bizzozeronii* have been detected in human patients [1,16,17,18,19]. The infection rate, however, is significantly lower compared to *H. pylori*, with reported rates of 0.2–6.0% in human patients suffering from gastric complains undergoing a gastric biopsy [17]. Nevertheless, these values may be an underestimation of the true prevalence, considering the lack of proper diagnostic tools [17].

Human gastric NHPH infection is usually accompanied by gastritis, antral erosions and duodenal ulcers [1,17,20]. Furthermore, NHPH infection has also been associated with gastric low-grade mucosa-associated lymphoid tissue (MALT) lymphoma, and the risk of developing this disease is higher than that with *H. pylori* infection [21]. Human infections with NHPH most likely originate from direct or indirect contact with infected animals. Living in close proximity with animals is therefore considered a major risk factor. Besides frequency, the intensity of animal contact is also important. Indeed, a higher incidence of these infections has been noted in farmers, staff of slaughterhouses, pet owners and children having intense contact with pets. Other transmission routes might be consumption of contaminated water and, for *H. suis,* consumption of contaminated pork [22,23,24]. Recent studies also demonstrated that patients infected with NHPH may be co-infected with *H. pylori* and/or another gastric *Helicobacter* species [12,25].

*H. pylori* infection rate is related to socioeconomic status, level of urbanization, sanitation, access to clean water and hygiene levels. This may explain why higher numbers of cases have been reported in developing countries (almost 80.0% on Africa), compared with low levels in developed countries (p.e. 24.4% on Oceania) [3]. At the individual level, several risk factors for *H. pylori* infection have been proposed, including older age, smoking, untreated water intake, alcohol and salty food consumption, and family history of gastric disorders [26]. It can be concluded that the disease outcome of *Helicobacter* spp. infection is a result of a complex interaction between host, environmental and bacterial factors [4].

In Europe, the overall prevalence of *Helicobacter* is around 47.0%, but in Portugal it can be as high as 86.4% [3]; for this reason, this country was selected for this study. Therefore, the aims of this research were: (1) to determine the occurrence of gastric *Helicobacter* species in a subset of patients treated at Hospital de São João, Porto, Portugal; and (2) to describe the outcome of the *Helicobacter* eradication treatment in those patients, and to determine their current *Helicobacter* infection status.

## 2. Materials and Methods

### 2.1. Patients Samples

Gastric samples were collected from Portuguese patients who underwent gastric resection as a treatment for gastric cancer (GC) (*n* = 17) or who underwent laparoscopic sleeve gastrectomy for morbid obesity (OB) (*n* = 63). In patients diagnosed with GC, two samples were collected, one from tumor tissue and one of adjacent tissue. In OB patients, three samples were collected from the gastric body/fundus region. Regardless of the origin, each specimen was divided in two fragments: one was fixed in 10% formalin for histological evaluation and the other was immediately frozen at −80 °C for further molecular studies. Samples were collected between March 2017 and July 2020, from surgeries at Centro Hospitalar Universitário de São João (CHUSJ), Porto, Portugal. All the patients included in the study were informed about the project and their written consent was requested. During the pre-surgical consultation, the medical doctor performed a small and anonymous survey, concerning personal details (age and gender), information regarding putative animal contact (frequency and type of animals), and evidence of a possible *Helicobacter* eradication treatment carried out in the 18 months prior to the gastric surgery. Information regarding the *Helicobacter* eradication treatment was confirmed with the clinical report from the hospital. None of the participants were considered vulnerable individuals. All the procedures were approved by the Ethics Committee from the CHSJ/FMUP (CES 127-15).

### 2.2. Histological Analysis of Helicobacter Species in Human Gastric Samples

Samples were fixed in 10% phosphate-buffered formalin and embedded in paraffin. Serial sections of 3 µm were made, both for haematoxylin-eosin (H&E) staining for histopathological evaluation and for immunohistochemical examination.

Sections stained with H&E were examined by a pathologist in order to confirm the gastric anatomical region. Additionally, a modified-Giemsa (MG) stain was performed to assist in the identification of *Helicobacter* spp. The bacterial density colonization was quantified according to the following classification: −, absence of organisms; +, few organisms (< 10 organisms/400×); ++, moderate number of organisms (10 to 50 organisms/400×); +++, large number of organisms (> 50 organisms/400×), as previously described [27].

### 2.3. Immunohistochemical Detection of Helicobacter Species in Human Gastric Samples

For the immunohistochemical (IHC) study, sections were deparaffinized, hydrated, and antigen retrieval was performed in 10 mmol/L sodium citrate buffer (pH 6.0, 10 min). Slides were cooled for 20 min at room temperature and rinsed twice in phosphate buffered saline with 0.05% Tween 20 (PBS-T 0.05%) for 5 min. After blocking endogenous peroxidase with 3% hydrogen peroxide in methanol for 10 min, slides were blocked for 30 min with normal goat serum (Dako, Santa Clara, CA, USA), diluted 1:5 in 10% bovine serum albumin (BSA 10%). Thereafter, slides were incubated overnight at 4 °C with a primary polyclonal antiserum against *H. pylori* (RBK012; Zytomed, Berlin, Germany), diluted 1:150 in BSA 5%, known to present immunoreactivity with a wide range of bacteria belonging to the *Helicobacter* genus [28]. Sections were rinsed with PBS-T 0.05% and incubated with secondary antibody goat anti-rabbit diluted 1:200 in BSA 5%, for 30 min (Dako, Santa Clara, CA, USA). Slides were rinsed again and incubated with avidin-biotin-peroxidase complex solution for 30 min (Vectastain^®^ Elite ABC HRP Kit, PK-6100, Vector Laboratories, Burlingame, CA, USA). Colour was developed with 3,3α-diamino-benzidine (DAB; Sigma, St. Louis, MO, USA) for 5 to 7 min at room temperature. Sections were then counterstained with haematoxylin, dehydrated and mounted. Slides were observed at light microscopy and positive immunoreactivity was recorded as a distinct golden-brown labelling of the bacteria located at mucosal surface, in gastric pits or glands and in parietal cells. Again, the amount of bacteria was evaluated according to the same classification described on the section above [27].

### 2.4. DNA Extraction and Helicobacter Species Identification through PCR Analysis

Cryopreserved gastric samples were homogenized, and DNA was extracted using the ExtractMe DNA Tissue Plus Kit (EM04, Blirt, Gdańsk, Poland), according to the manufacturer instructions.

The primers *Hcom1* and *Hcom2* (Table S1) were used to amplify a 390-bp fragment of the *16S* rRNA gene sequence to determine the presence of bacteria belonging to the *Helicobacter*-genus [29,30,31]. PCR was performed in reaction volumes of 20 µL containing 1.5 mM MgCl_2_ (Promega), 1x GoTaq^®^ Flexi PCR buffer (Promega, Madison, WI, USA), 200 µM deoxynucleotide triphosphates (dNTPs) (Bioline, Meridian Bioscience, Ohio, USA), 0.5 µM forward primer, 0.5 µM reverse primer, 1U GoTaq^®^ Flexi DNA polymerase (Promega) and 1 µL of the DNA extract. PCR amplification was performed under the following conditions: 5 min of preincubation at 94 °C, followed by 40 cycles of 1 min at 94 °C, 1 min at 63 °C, and 1 min at 72 °C. A final extension was performed for 5 min at 72 °C. As a positive control, DNA extracted from a pure culture of *H. suis* (HS5) was used [32].

*Helicobacter* species-specific PCR assays were performed for *H. suis, H. bizzozeronii, H. felis, H. salomonis, H. heilmannii, H. ailurogastricus* and *H. pylori.* The primers used for each *Helicobacter* species-specific PCR and the respective amplification conditions are listed in Table S1. PCR was performed in reaction volumes of 20 µL containing 2.5 mM MgCl2 (Promega), 1x GoTaq^®^ Flexi PCR buffer (Promega, Madison, WI, USA), 200 µM deoxynucleotide triphosphates (dNTPs) (Bioline), 0.5 µM forward primer, 0.5 µM reverse primer, 0.6U GoTaq^®^ Flexi DNA polymerase (Promega), and 1 µL of the DNA extract. DNA extracted from a pure culture of *H. suis* (HS5) was used as the positive control.

Five microlitres of each PCR amplification product was analysed through gel electrophoresis in 1.5% agarose (AGRMP-RO Roche, Merck KGaA, Darmstadt, Germany) in TBE buffer (VWR Life Science, Amsterdam, The Netherlands). GeneRuler 100bp Plus DNA Ladder (Thermo Scientific™ SM0323) was used as a weight marker. Images were acquired on a UV transilluminator (UVP PhotoDoc-it Imaging Systems, Fisher Scientific, Hampton, NH, USA).

Sequencing analysis of samples showing a positive PCR result upon genus- and/or species-specific PCR was performed in order to confirm the *Helicobacter* species present. Sequencing analysis of amplicons positive for *Helicobacter* genus-specific PCR allows discrimination between *H. suis,* canine- and feline-associated gastric NHPH as a group, and *H. pylori.* Sequencing was done at Eurofins Genomics^®^ (Edersberg, Germany). All obtained sequences were processed using the BioNumerics^®^ software (version 7.6.3, Applied Maths, Sint-Martens-Latem, Belgium). Finally, the aligned sequences were blasted using the NCBI Nblast tool (https://blast.ncbi.nlm.nih.gov/Blast.cgi; last accessed on 12 May 2021). A cut-off value of 96% was used for average nucleotide identity as a threshold for species delineation [33].

A patient was considered positive in the case that at least one of the samples taken was positive in at least one of the PCR assays, followed by a confirmatory sequencing result (not based on histology results).

## 3. Results

### 3.1. Patients Data

A total of 221 gastric samples belonging to 80 patients were evaluated in this study, comprising 34 samples obtained from 17 GC patients and 187 samples from 63 OB patients. The mean age was 50.9 ± 14.8 years (72.5 ± 10.0 for the GC patients and 45.1 ± 9.6 for the OB patient). The female:male ratio for the overall patients evaluated was 1.8:1.

### 3.2. Histological and Immunohistochemical Detection of Helicobacter Species in Human Gastric Samples

Histological analysis using H&E staining confirmed the presence of malignant epithelial neoplasia in tissue fragments from GC patients, while gastric samples from OB patients showed no signs of neoplastic transformation.

**Figure 1:**
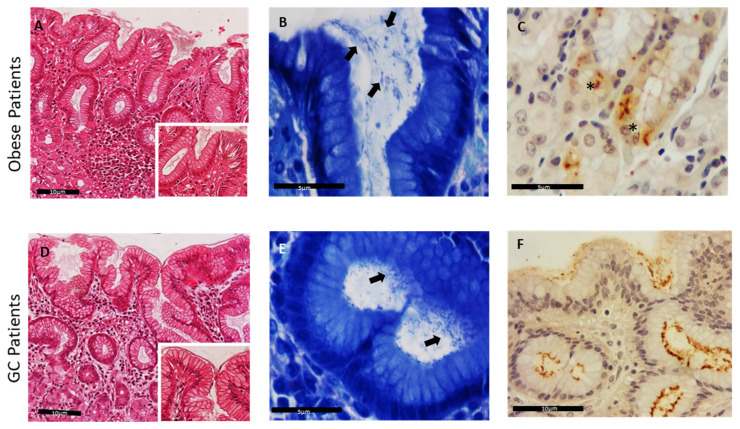
Detection of *Helicobacter* species in human gastric mucosa sections. H&E stain of gastric sections from obese (OB) patients (**A**) and gastric cancer (GC) patients (**D**), 200× and 400× (inset). Modified-Giemsa stain, highlighting bacteria at the epithelium surface and gastric crypts (black arrows), in obese patients (**B**) and GC patients (**E**), magnification 600×. (**C**,**F**) *Helicobacter* spp. immunopositivity within the superficial mucus, in the lumen of gastric deep glands, 400×, and inside parietal cells, 600×, (*) in obese patients (**C**) and GC patients (**F**).

Based on the MG stain, bacteria were detected in 43 out of 80 patients (53.8%). In more detail, *Helicobacter* spp.-like organisms were visualized in 7 out of 17 GC patients (41.2%) and in 36 out of 63 OB patients (57.1%) (Table 1). These organisms were mainly identified within the mucus layer and close to the superficial gastric epithelium (Figure 1A–D). The results of histological and immunohistochemical analysis are presented on Figure 1 and Table 1.

Using IHC, curve- or spiral-shaped bacteria were identified in 53 out of 80 patients (66.3%). In the GC cases, *Helicobacter* spp. immunopositivity was detected in 9 of the 17 patients (52.9%), while in the remaining 8, no specific nor compatible immunoreactivity was identified (47.1%). Among the samples of OB patients, *Helicobacter* spp. immunopositivity was detected in 44 out of 63 patients (69.8%), whereas 19 individuals were considered negative (30.2%). Both MG stain and IHC technique allowed the identification of bacteria, not only when they were present in smaller amounts (+/++), but also when present in more discrete locations such as the lumen of deeper glands and the cytoplasm of parietal cells (Figure 1, Table 1).

### 3.3. Detection of Helicobacter Species

*Helicobacter* genus-specific PCR was performed in all human gastric samples, followed by sequencing of the positive amplicons in order to discriminate between *H. suis, H. pylori* and the group of canine- and feline-associated gastric NHPH. PCR results were positive in 75 samples (33.9%), of which one proved false positive based on sequencing analysis of the amplicon and the remaining 74 were classified as *H. pylori*. This corresponded to presence of *H. pylori* in at least one sample of 37 out of 80 patients (46.3%) (Table 1 and Table S2). These included 2 out of 17 GC patients (11.8%) and 35 out of 63 OB patients (55.5%).

Species-specific PCR assays for *H. suis, H. bizzozeronii, H. felis, H. salomonis, H. heilmannii, H. ailurogastricus* and *H. pylori* were performed in all human gastric samples, again followed by sequencing of the positive amplicons in order to confirm presence of the *Helicobacter* species in question. In 30 samples (13.6%), PCR results for *H. pylori* were found positive, of which one proved false positive based on sequencing analysis of the amplicon, while in the remaining 29, the presence of *H. pylori* was confirmed. This corresponded to presence of *H. pylori* in at least one sample of 17 out of 80 (21.3%) (Table 1 and Table S2), with the majority of these patients belonging to the OB group (15 patients, 23.8%), and two patients belonging to the GC group (11.8%). All samples found positive based on the species-specific PCR assay for *H. pylori* were also found positive for *H. pylori* based on the genus-specific PCR assay. As for *H. suis*, two samples (0.9%) were found to be positive, with the PCR result again confirmed by sequencing analysis of the amplicon. These two samples belonged to two different patients (2.5%) in whom the presence of *H. pylori* was also detected based on the genus-specific PCR assay and who belong to the OB group (2 out of 63; 3.2%). Results of all other species-specific PCR assays performed were negative.

Comparisons between the different techniques used for the identification of gastric *Helicobacter* species demonstrated that 24 patients (30.0%) were positive in all the techniques used (MG, IHC and PCR + sequencing). On the other hand, in 24 other patients (30.0%) a positive result was obtained with MG or IHC, but the samples were negative for genus- and/or species-specific PCR. Furthermore, six patients (7.5%) were positive with PCR + sequencing, and negative with other techniques used.

Detailed results are listed in Table 1 and Table S2.

There was no correlation between gender and presence of *Helicobacter* species in this cohort of patients.

### 3.4. Association between Helicobacter spp. Presence and Animal Contact

Among the 80 patients included in the study, 68 answered the survey (85.0%), of which 13 belong to the GC group (13 out of 17; 76.5%) and 55 to the OB group (55 out of 63; 87.3%). Close contact with animals was referred to by 55 patients (80.9%), comprising 11 out of 13 GC (84.6%) and 44 out of 55 OB patients (80.0%). The great majority of the respondents classified the contact with pets as a regular and daily basis (51/55; 92.7%). Close contact with dogs and cats was mentioned by 8 out of 11 GC patients (72.7%) and 43 out of 44 OB patients (97.7%). Contact with other animals, such as chickens, rabbits, parakeets, sheep, etc., was also mentioned by 34.5% of the patients, including 6 out of 11 GC (54.5%) and 13 out of 44 OB patients (29.5%).

**Figure 2:**
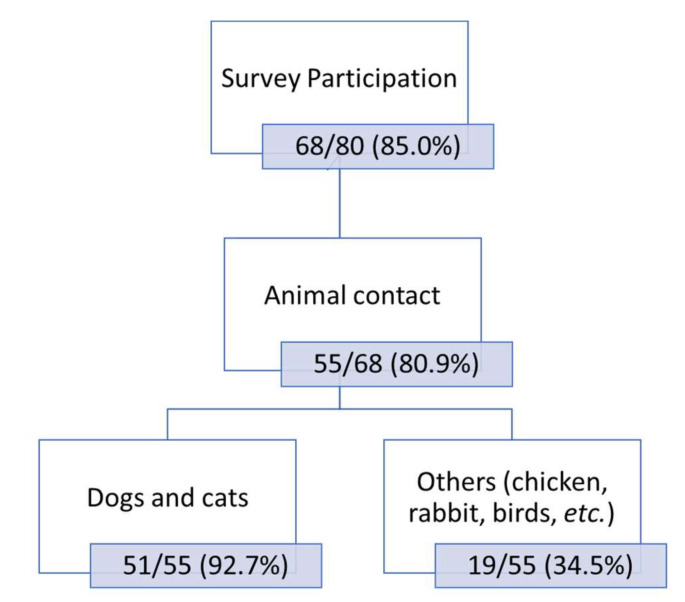
Overall results of the animal contact based on the patients’ surveys.

All the information obtained from the inquiries is resumed in Figure 2.

### 3.5. Helicobacter spp. Presence in Patients with Previous History of Eradication Treatment

**Figure 3:**
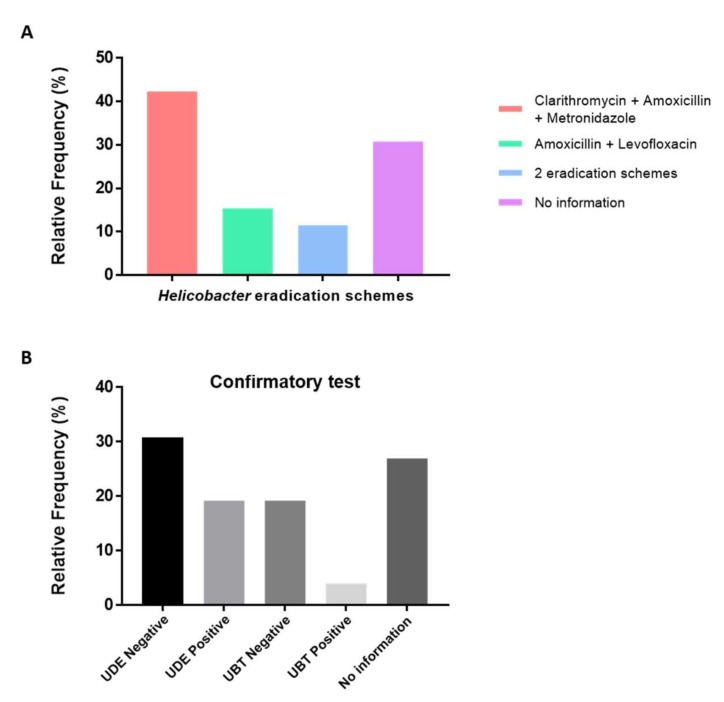
*Helicobacter* eradication treatment. (**A**) Detailed information about the therapeutical schemes used. (**B**) Confirmatory test performed to evaluate the presence of *Helicobacter* organisms after eradication treatment. UDE: upper digestive endoscopy; UBT: urea breath test.

**Figure 4:**
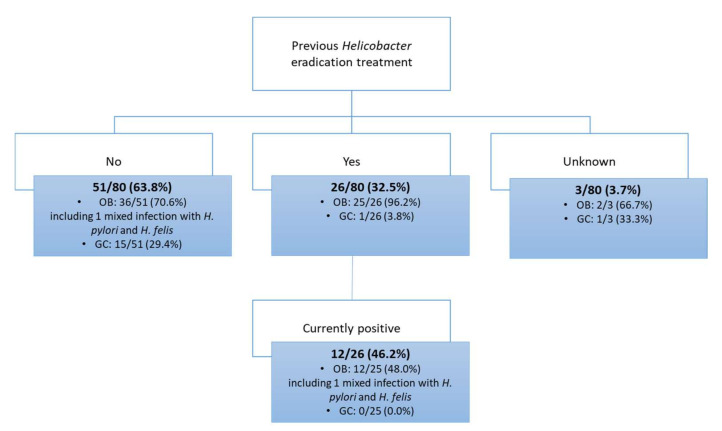
Information about the *Helicobacter* eradication treatment and the current infectious status.

Information regarding previous *Helicobacter* eradication therapy in the last 18 months was collected, both from the surveys and clinical reports. Results indicated that 26 out of 80 patients (32.5%) were subjected to eradication treatment, 51 out of 80 (63.8%) did not receive any treatment, and for three patients (3.7%), this information was not available (Figure 3 and Figure 4).

Regarding the *Helicobacter* eradication scheme, the triple standard therapy (proton pump inhibitor (PPI), metronidazole, clarithromycin/amoxicillin) was the most common scheme performed (11 out of 26; 42.3%). Amoxicillin and levofloxacin were used in 4 out of 26 patients (15.4%), and three patients (11.5%) were subjected to two different *Helicobacter* eradication protocols (first with the triple standard therapy and later with amoxicillin and levofloxacin). For the remaining eight patients, there was no information available regarding the treatment performed (Figure 3).

The patients subjected to the eradication treatment belonged predominantly to the OB group (25 out of 26 patients; 96.2%). Amongst these 25 patients, results indicated that *Helicobacter* organisms were still detected in 12 individuals (48.0%). Based on genus- and species-specific PCR and sequencing of the amplicon, a mixed infection with two *Helicobacter* spp. (*H. pylori* + *H. felis*) was found in one of those 12 patients (Figure 3 and Figure 4). The other OB patient presenting a mixed infection with *H. pylori* and *H. felis* was not subjected to any *Helicobacter* eradication scheme in the 18 months prior to the gastric surgery.

In the GC group, only one patient out of 26 received an eradication treatment prior to surgery (3.8%), and in this particular individual no *Helicobacter* DNA was detected.

For 26 out of 80 patients (32.5%), a confirmatory test was also performed after the eradication treatment, either by urea breath test (UBT) (6 out of 26; 23.1%) or using other methods (upper digestive endoscopy (UDE) (13 out of 26; 50.0%)). For those evaluated through UBT, five individuals (19.2%) were negative for urease-producing organisms and only 1 out of 26 was positive (3.9%) (Figure 3). The UDE screening demonstrated that 8 out of 26 (30.8%) patients were negative for *Helicobacter*, while in 5 out of 26 individuals (19.2%), the bacteria were still detected by species-specific PCR followed by sequencing of the amplicon. For seven individuals (26.9%), there was information that a confirmatory test was performed, but the result was not mentioned.

All the information is presented in Figure 3 and Figure 4.

## 4. Discussion

In this study, human gastric samples were collected from 17 GC and 63 OB patients and screened for the presence of *Helicobacter* spp. Since *Helicobacter* organisms are not easily visualized with H&E, two additional staining techniques, namely MG and IHC, were used to highlight their presence [27,28]. The percentage of *Helicobacter-*positive patients based solely on MG and IHC analysis was 53.8% and 66.3%, respectively. Based on the IHC analysis, bacteria were identified in the mucus layer and superficial gastric epithelium, as well as within deep glans and even in the cytoplasm of the gastric parietal cells. The canaliculi of parietal cells are characterized by an extreme acidic pH due to the secretion of hydrochloric acid, that could lead to possible unspecific immunoreactivity of the antibody used in IHC. The use of electronic microscopy could be a good approach to confirm the presence of *Helicobacter* species inside the parietal cells, although this was not performed in our study. It has been suggested that *H. pylori* may present an intracellular location representing one of the few bacteria able to invade the host cell membrane, and being internalized in gastric epithelial or immune cells [34]. Interestingly, the presence of NHPH inside parietal cells was already described in the canine and feline gastric mucosa and it was thought that this phenomenon was an exclusive feature of NHPH [28,35,36]. Although IHC does not allow differentiation between the different gastric *Helicobacter* spp., in most patients only *H. pylori* was detected through PCR. This indicates that *H. pylori* could also be able to invade parietal cells. Intracellular localization might be a strategy used by the bacteria to evade to the immune system, and to resist to the eradication treatment.

On the other hand, higher sensitivity for *Helicobacter* spp. detection using molecular techniques, such as PCR, is recognized [37] allowing the identification of the bacteria at the genus or species level, depending on the primers used [27]. In our study, *Helicobacter* genus-specific PCR followed by sequencing revealed the presence of *H. pylori* DNA in 46.3% of the patients, while this was only verified in 21.3% with the species-specific PCR assay for *H. pylori.* This suggests that the sensitivity of the species-specific PCR assay used here is lower than that of the genus-specific assay in regard of *H. pylori* detection. This could be due to many reasons, such as: (1) not yet identified/uncultured NHPH species with high similarity to *H. pylori* are being detected with the genus-specific PCR; and/or (2) some of the already identified NHPH species showing a high genome similarity to *H. pylori,* for which we did not perform a species-specific PCR, can give some false positive results.

In contrast, our results showed a lower detection of *Helicobacter* species through PCR in comparison to MG or IHC. This can be explained by the high specificity of the molecular techniques. The genus- and species-specific PCR followed by sequencing of the positive amplicons is a more accurate and precise technique for identification of these *Helicobacter* species showing high similarity. On the other hand, the identification based only on histological techniques can lead to false positive results since it depends on human evaluation and screening, and other bacterial species can also be stained and interfere with the result. Furthermore, with these techniques we cannot identify the bacteria at species level, which is why the PCR followed by sequencing was considered the best strategy for the detection of bacterial DNA in the human gastric samples. To this end, herein, a conventional *Helicobacter* genus-specific PCR assay was conducted based on the *16S* rRNA gene which was able to discriminate between *H. suis, H. pylori* and the group of canine- and feline-associated gastric NHPH upon sequencing, in addition to species-specific PCR analysis for *H. suis, H. bizzozeronii, H. felis, H. salomonis, H. heilmannii, H. ailurogastricus* and *H. pylori*.

Infection of the human gastric mucosa with *H. pylori* leads to chronic inflammation, characterized by infiltration of neutrophils and macrophages [4,7,38,39]. The density of bacterial colonization by *H. pylori* is reported to be high in the initial steps of infection, but it decreases during the disease progression, being almost devoid of organisms in late stages of cancer transformation that are characterized by a marked increase of *Lactobacillus* composition [40,41]. However, even in these extreme conditions, *H. pylori* was able to survive and persist, being detected in 11.8% of the GC patients. Regarding the OB group, *H. pylori* was found in 55.5% of the patients and we can hypothesize that this high rate can be related to less extreme gastric microenvironment conditions in comparison to those patients suffering from gastric carcinoma. Furthermore, based on species-specific PCR followed by sequencing of the amplicon, two patients exhibited mixed infections with two *Helicobacter* species, namely *H. pylori* and the canine- and feline-associated *H. felis* (2.5%), confirming that mixed infections with *H. pylori* and animal-associated and zoonotic gastric NHPH may occur [12,25].

Although these patients did not present any clinical alteration besides the obesity condition, histopathological analysis demonstrated that the majority presented signs of hyperplasia of the superficial epithelium, gastritis and presence of multiple lymphoid follicles (data not shown). These findings could be related with the presence of *H. pylori* [42].

The low rate of NHPH occurrence (2.5%) goes towards the values described in the literature [17]. Amongst the patients who answered the survey, the great majority reported a close and frequent contact with different animal species (80.9%). Interestingly, one of the patients presenting mixed infection with *H. pylori* and *H. felis*, referred to a close contact with a cat as a pet, while the other patient reported no contact with animals. These results confirm that contact with pets does not necessarily result in a NHPH infection, raising questions about how humans get infected with these *Helicobacter* species.

The efficacy of the *Helicobacter* eradication treatment has been declining in the past decade, due to several factors such as side effects, increasing antibiotic resistant strains, and the cost of the antibiotic regimens [11]. Acquired resistance to antibiotics has been described not only for *H. pylori*, but also for some NHPH isolates, such as *H. heilmannii, H. ailurogastricus* and *H. suis* [13,15]. Since these bacteria can be found in the stomach of several animals and also in humans, the presence of acquired resistance to some antimicrobial compounds can have an impact both on human and veterinary health. Among the 80 individuals included in this study, 32.5% were subjected to eradication treatment in the last 18 months. Remarkably, in 46.2% of these patients, *Helicobacter* spp. (including one of the patients with a mixed *H. pylori + H. felis* infection) were still detected in gastric tissues. There are several factors that might explain this finding: (1) the treatment was not strictly carried out by the patient (incomplete or unfinished) or was not effective at all; (2) the treatment was carried out too close to the confirmatory test, so there had not yet been an opportunity for the bacteria to be completely eliminated; or (3) the patient was reinfected after the treatment. Among the patients who remained *Helicobacter*-positive after treatment, the triple standard therapy was the most frequent scheme, followed by treatment with a combination of amoxicillin and levofloxacin; some patients were even submitted to two different eradication schemes. After the eradication treatment, patients should be submitted to a confirmatory test, either by urea breath test or by other techniques that usually require endoscopy to confirm the elimination of the bacteria. Previously performed confirmatory tests indicated a negative *Helicobacter* result in four patients, that later were found infected with *H. pylori* (through PCR and confirmed by sequencing), which calls into question the efficacy of the treatment.

Portugal has been considered one the countries with the highest resistance percentage to clarithromycin (42.35%), one of the main antibiotics that constitute the standard triple therapy [43]. This could partially justify the relative low efficiency of the eradication protocol, since 42.3% of the patients were submitted to a therapeutical scheme based on this drug.

Additionally, limitations of the study have to be taken into account. Different surgical approaches were used to collect the gastric samples according to the type of patients (cancer vs. obese) which may have influenced the results obtained. Addition of a control group would certainly benefit comparisons and statistical analysis. Moreover, with respect to *Helicobacter* spp. eradication treatment, the results described were due to different therapeutical schemes using different drugs, and part of the information herein obtained was based on the testimony and memory of the patients themselves.

## 5. Conclusions

In conclusion, the current results confirm that mixed infections with at least two *Helicobacter* spp. can occur in humans. Therefore, it can be advised to test for the presence of *H. pylori* as well as gastric NHPH. Additionally, due to the decreased efficiency of the current *Helicobacter* eradication treatment, the development of new therapeutical strategies is necessary.

## Figures and Tables

**Table 1:** Occurrence of *Helicobacter* species in human gastric samples, based on different technical approaches (MG: modified-Giemsa stain; IHC: immunohistochemistry; PCR: polymerase chain reaction). Detailed information about each patient is present in Table S2.

Gastric Samples	Detection Methods Positive (Percentage and Number)				
	MG	IHC	Genus-Specific PCR + Sequencing	Species-Specific PCR + Sequencing	
				*H.* *pylori*	*H* *. felis*
Total (*n* = 80)	53.8% (43/80)	66.3% (53/80)	46.3% (37/80)	21.3% (17/80)	2.5% (2/80)
Gastric cancer patients (*n* = 17)	41.2% (7/17)	52.9% (9/17)	11.8% (2/17)	11.8% (2/17)	-
Obese patients (*n* = 63)	57.1% (36/63)	69.8% (44/63)	55.5% (35/63)	23.8% (15/63)	3.2% (2/63)

## Supplementary Materials

The following supporting information can be downloaded at: https://www.mdpi.com/article/10.3390/jpm12020181/s1. Table S1: Oligonucleotide primers used for PCR amplification. Table S2: Different approaches for *Helicobacter* species detection in human gastric samples (Sequencing, modified-Giemsa stain, IHC, PCR).
