# Supplementary material for: Presence of Helicobacter Species in Gastric Mucosa of Human Patients and Outcome of Helicobacter Eradication Treatment
Source: J Pers Med. 2022 Jan 29;12(2):181. doi: 10.3390/jpm12020181 (PMC8879780; doi:10.3390/jpm12020181)
Supplement: Supplementary file 1 [file jpm-12-00181-s001.zip › Matos et al. Supplementary table S1.pdf]

Table S1. Oligonucleotide primers used for PCR amplification.

Assay		Primer	Sequence (5’-3’)	Target gene - Amplicon size (bp)	PCR Conditions
***Helicobacter* genus-specific PCR assay**		Hcom1	GTA AAG GCT CAC CAA GGC TAT	*16S rRNA* - 390	5' 94°C<br>40x (1’ 94°C-1’ 63°C-1’ 72°C)<br>5' 72°C
		Hcom2	CCA CCT ACC TCT CCC ACA CTC		
***Helicobacter* species-specific PCR assays**	*H. suis*	BFHsuis_F1	AAA ACA MAG GCG ATC GCC CTG TA (M = A/C)	*UreAB* - 150	3’ 95°C<br>40x (1' 94°C-1' 59°C-1' 72C)<br>5’ 72°C
		BFHsuis_R1	TTT CTT CGC CAG GTT CAA AGC G		
	H. bizzozeronii	BFHbizz_F1	CGC TTT GAA CCC GGT GAG AAA A	*UreAB* - 172	4' 95°C<br>45x (1' 94°C-1' 58°C-1' 72C)<br>10' 72°C
		BFHbizz_R1	TAT CGC AAC CGC AAT TCA CAA CA		
	H. felis	BFHfel_F2	GCT GGT GGC ATC GAT ACG CAT	*UreAB* - 154	4' 95°C<br>45x (30" 94°C-30" 60°C-1' 72°C)<br>10' 72°C
		BFHfel_R2	TTT TTA GAT TAG CGC GTC CGG GA		
	H. salomonis	Hsal_FQ_PAR	CTC TTA TGA GTT GGA CTT GGT GCT CAC CAA T	*UreAB* - 91	4' 95°C<br>45x (30" 94°C-30" 61°C-1' 72°C)<br>10' 72°C
		Hsal_RQ_PAR	TTT GCC ATC TTT AAT TCC AAT GTC GGC		
	H. heilmannii	Hh-IceA-FWQ	GTT TCC AAC CAA AAG ACT CA	*IceA* - 135	5' 94°C<br>45x (30" 94°C-30" 55°C-30" 72°C)<br>10' 72°C
		Hh-IceA-RVQ	ATT GCC TAG AGG TTG TGT TG		
	H. ailurogastricus	Ha-LpsA-FWQ	CTT GAG TAC GGC GAT GTC AAT	*LpsA* - 136	5' 94°C<br>45x (30" 94°C-30" 55°C-30" 72°C)<br>10' 72°C
		Ha-LpsA-RVQ	GGG GAA AAA TGT GCT TGA AGT		
	H. pylori	BFHpyl_F1	AAA GAG CGT GGT TTT CAT GGC G	*UreAB* - 217	4' 95°C<br>45x (30" 94°C-30" 59°C-1' 72°C)<br>10' 72°C
		BFHpyl_R1	GGG TTT TAC CGC CAC CGA ATT TAA		
